# Relationship Between Unmet Social Needs and Care Access in a Veteran Cohort

**DOI:** 10.1007/s11606-023-08117-3

**Published:** 2023-06-20

**Authors:** Deborah Gurewich, Amy M. Linsky, Kimberly L. Harvey, Mingfei Li, Ida Griesemer, Risette Z. MacLaren, Rory Ostrow, David Mohr

**Affiliations:** 1 Center for Healthcare Organization and Implementation Research (CHOIR), VA Boston Healthcare System, Boston, MA USA; 2 Section of General Internal Medicine, Boston University School of Medicine (BUSM), Boston, MA USA; 3 Section of General Internal Medicine, VA Boston Healthcare System, Boston, MA USA; 4 CHOIR, VA Bedford Healthcare System, Bedford, MA USA; 5 Department of Mathematical Sciences, Bentley University, Waltham, MA USA; 6 Department of Health Policy and Management, Boston University School of Public Health, Boston, MA USA

**Keywords:** social determinants of health, veterans, care access, adherence

## Abstract

**Background:**

The association between unmet social needs (e.g., food insecurity) and adverse health outcomes is well-established, especially for patients with and at risk for cardiovascular disease (CVD). This has motivated healthcare systems to focus on unmet social needs. Yet, little is known about the mechanisms by which unmet social needs impact health, which limits healthcare-based intervention design and evaluation. One conceptual framework posits that unmet social needs may impact health by limiting care access, but this remains understudied.

**Objective:**

Examine the relationship between unmet social needs and care access.

**Design:**

Cross-sectional study design using survey data on unmet needs merged with administrative data from the Veterans Health Administration (VA) Corporate Data Warehouse (September 2019–March 2021) and multivariable models to predict care access outcomes. Pooled and separate rural and urban logistic regression models were utilized with adjustments from sociodemographics, region, and comorbidity.

**Subjects:**

A national stratified random sample of VA-enrolled Veterans with and at risk for CVD who responded to the survey.

**Main Measures:**

No-show appointments were defined dichotomously as patients with one or more missed outpatient visits. Medication non-adherence was measured as proportion of days covered and defined dichotomously as adherence less than 80%.

**Key Results:**

Greater burden of unmet social needs was associated with significantly higher odds of no-show appointments (OR = 3.27, 95% CI = 2.43, 4.39) and medication non-adherence (OR = 1.59, 95% CI = 1.19, 2.13), with similar associations observed for rural and urban Veterans. Social disconnection and legal needs were especially strong predictors of care access measures.

**Conclusions:**

Findings suggest that unmet social needs may adversely impact care access. Findings also point to specific unmet social needs that may be especially impactful and thus might be prioritized for interventions, in particular social disconnection and legal needs.

**Supplementary Information:**

The online version contains supplementary material available at 10.1007/s11606-023-08117-3.

## BACKGROUND AND OBJECTIVE

Access is a concept concerned with the degree to which individuals can obtain needed services from the medical care system to achieve the best possible outcome.^1^ Some indicators of care access barriers focus on unrealized opportunities to use services known to have measurable effect.^2–4^ In this study, we focus on one such measure (missed appointments, an established care access metric) and a related measure (medication non-adherence), a potentially novel access metric that fills a gap in the current lexicon of access metrics given the role of access to and use of prescribed medications in contributing to health.^5–7^ We focus on these measures through the lens of unmet social needs (e.g., food insecurity, loneliness).

The association between unmet social needs (hereafter: “social needs”) and adverse health outcomes is well-established, especially for patients with and at risk for cardiovascular disease (CVD).^8–10^ The American Heart Association declared that the most significant opportunities for improving CVD outcomes lie with addressing the social determinants of cardiovascular disease.^11^ Yet, little is known about the mechanisms by which social needs impact health, which by extension limits how and where to target interventions and evaluate their success.^12^ One conceptual framework posits that social needs may impact health by adversely impacting care access.^12^ For example, a patient who is food insecure may prioritize her limited funds for buying food, a necessity, instead of paying the co-payment for her hypertension medication or a patient may miss a primary care visit for his diabetes if he is unable to solve his transportation needs.^13,14^ In turn, medication non-adherence and missed appointments (also referred to as “no-shows”) are associated with poor health outcomes.^15,16^

The relationship between social needs and access is understudied. Social needs, most especially transportation needs, have been associated with no-show visits, but studies to date are relatively scant and reliant on convenience and in most instances small samples, thus limiting generalizability. ^10,13,17–19^ Food insecurity and housing needs are reported to increase the risk of medication non-adherence but less is known about other social needs, such as social disconnection, transportation needs, and unemployment.^20–22^ A recent systematic review of studies examining the relationship between social needs and medication adherence identified relatively few studies and among studies included, key limitations noted included reliance on self-report to measure medication adherence (i.e., relatively few studies used an objective measure such as administrative claims) and failure to evaluate the effect of two or more social needs.^22^

These knowledge gaps are especially relevant for the Veterans Health Administration (VA). The criteria prioritizing access to VA services to those with financial need result in many VA-enrolled Veterans having low incomes, and thus high risk for social needs.^23,24^ However, we identified only one study examining the relationship between social needs and care access among Veterans, finding that social support was not associated with medication non-adherence.^14^ Additionally relevant to the VA is that one-third of VA-enrolled Veterans reside in rural areas. Rural compared to urban America is associated with higher poverty rates, fewer social services,^25–27^ and more complex logistics in accessing resources. For these reasons, the relationship between social needs and care access may be especially significant for rural populations. Yet, as far as we know, there are no studies focusing on social needs in rural populations, let alone rural Veterans.

In this study, we evaluated the relationship between social need burden and care access among a national stratified random sample of rural and urban Veterans with and at risk for CVD, as well as variation in these relationships between specific social needs and care access. Secondary analyses assessed whether these relationships differed for rural and urban Veterans. This study was part of a larger study designed to develop interventions to reduce disparities among rural Veterans with and at risk for CVD.

## DESIGN AND SUBJECTS

The study used a cross-sectional design consisting of primary data collected through a mail survey (fielded 09/1/2020–12/31/2020) combined with administrative data from the VA Corporate Data Warehouse (CDW) (9/1/2019–03/31/2021). The study was deemed exempt by the VA Boston Healthcare System Institutional Review Board.

Using CDW data, we first identified our sampling frame (approximately 4 million unique patients) based on patients with select diagnostic (ICD10) codes over a 15-month period (01/01/19–03/24/20). We also conducted a power analysis to estimate the minimum sample size needed for a logistic model (*N* = 1472) (see Supplemental File 1).

We then applied stratified random sampling based on 3 criteria to create 24 strata: (1) cohort (CVD vs. CVD-risk; (2) region (Continental, Northeast, Pacific, and Southeast); and (3) race (White, Black, and other).^28^ Other race included Asian, Native Hawaiian/Pacific Islander, and American Indian/Alaskan Native. We oversampled smaller stratum to have at least 86 potential respondents, which ensured a minimum final sample per stratum of *n* = 30 (assuming a 35% response rate). This yielded a final survey sample of 5204 patients. We fielded the survey from September to December 2020, mailing an initial postcard notice, followed by the survey, and then, among non-responders, a reminder postcard, and as needed, a second survey.^29,30^

## MAIN MEASURES

### Outcome Measures

For medication non-adherence, we examined medications dispensed during the study period (with an additional 90-day look-back prior to the 9/1/2019 study start date to account for medications dispensed earlier that could still be in the patient’s possession). We selected medication classes typically prescribed to treat CVD and at-risk CVD conditions, including antilipemic, antiglycemic, ACE inhibitors, angiotensin II inhibitors, and select diuretics (Supplemental Table 1).

We calculated the proportion of days covered (PDC) for all drugs dispensed within each drug class and then averaged the PDC across all medication classes to determine adherence.^31^ The measure captures the number of days with a medication in hand, accounting for the initial fill and all subsequent refills during the study period. Consistent with the literature, we dichotomized the measure so that patients with a PDC less than 80% were considered non-adherent.^32^

The no-show measure was computed based on the number of non-administrative, scheduled appointments that were coded as no-shows plus those canceled by the patient after the appointment date divided by the total number of appointments.^33^ We considered appointments made to primary care, mental health, and select specialty care clinics. The measure was calculated as a percentage ranging between 0 and 100% and then dichotomized (0 or 1) so that patients with a value greater than 0 were coded as having no-show appointments.

### Predictor Measure

The independent variables of primary interest were derived from the survey data, which consisted of 22 items, of which 10 items represented nine social needs (see Supplemental Table 2). Measures were selected from a variety of sources.^34–39^ Five measures asked about current need (housing, employment, finance, social disconnection, neighborhood safety) and four measures asked about need in the past 12 months (utility, transportation, legal, food). Item response options varied; thus, we dichotomized responses to indicate whether a respondent had a particular unmet need (0 or 1). We summed the total number of unmet needs among respondents (range 0 to 9). Based on the distribution, we coded the measure to include four categories based on the number of unmet needs: 0, 1, 2, 3, or more.

### Covariates

Based on research highlighting connections between other characteristics and social needs, we identified additional variables from VA CDW for use in our models.^40,41^ Covariates included sex (male or female); rural or urban setting based on Rural–Urban Commuting Area codes from the 2010 census defined and applied to patients; age in years; race (White, Black, other); Hispanic or non-Hispanic ethnicity; education (high school or less); and region.^28^ To account for health status, we used the Charlson Comorbidity Index (CCI; values of 0, 1, 2, or more). ^20,28^

We adjusted for VA priority status using level of service-connected disability and income: priority group 1 (50% or greater disability); groups 2 and 3 (10–50% disability); groups 4 and 5 (mix of disability and low income); and groups 6 through 8 (no service-connected disability or being above an income threshold requiring co-payments).^42^ We included in the medication non-adherence and no-show models respectively a continuous variable indicating the number of unique medications dispensed to the patient during the study period and the total number of outpatient visits of interest.

## ANALYSIS

We used descriptive statistics to describe the sociodemographic and clinical characteristics of our sample as well as the care access measures. We compared rural and urban sample members either through a means test or chi-square test. For each care access measure, we conducted a multivariate regression computing odds ratios (OR) and 95% confidence intervals (CI) through weighted logistic models. Each model included the social need count variable and the covariates described in the prior section. In supplemental analyses, we ran the same 2 models separately for rural and urban Veterans, identifying non-overlapping CIs. To understand the effects of specific social needs, we conducted fully adjusted models for each outcome measure that included all nine social needs (as being met or unmet) in lieu of the composite measure to obtain separate odds ratio point estimates and CIs for each unmet need predictor. As a sensitivity analysis for the no-show model, we ran additional models that adjusted the threshold no-show rate required to be coded as 1 using the following cut points: 0.00<  to 0.10, 0.10<  to 0.33, 0.33<  to 0.55, and 0.55< . All analyses were completed using SAS 9.2.^43^

## KEY RESULTS

### Descriptive Results

We obtained a response rate of 53.7% (*N* = 2801). Missing values on one or more variables yielded a final analytic sample of 2770 respondents. The average age of the respondents was 70 and consisted of 95% male, 85% White, 96% non-Hispanic or Latino, 63% with some education beyond high school, and 43% scoring 2 or more on CCI (Table 1). The average number of medications dispensed per patient was 2.3 and average number of visits was 3.1. Rural compared to urban Veterans were less likely to be female (3.8% vs. 6.4% respectively) and Black race (6.8% vs. 17.0% respectively); they were also on average dispensed more medications (2.5 vs. 2.2) but had fewer outpatient visits (2.8 vs. 3.3).

**Table 1:** Summary of Dependent and Independent Variable for Urban and Rural Veterans

Characteristics		Overall, *n* (%)	Urban, *n* (%)	Rural, *n* (%)	*p*-value
		(*n* = 2770)	(*n* = 1332)	(*n* = 1438)	
Dependent measures					
Medication adherence	<80%	752 (35.73)	365 (36.73)	387 (34.14)	.24
No-shows	≥1	1493 (55.28)	734 (57.75)	759 (51.34)	.002
Independent measures					
Age/years, *mean (SD)*		70.63 (11.27)	70.39 (11.17)	71.02 (10.73)	<.0001
No. medications, *median (IQR)*		2.29 (1.15, 3.83)	2.18 (1.11, 3.68)	2.48 (1.22, 4.04)	<.0001
No. visits, *median (IQR)*		3.10 (1.43, 5.60)	3.29 (1.51, 5.79)	2.83 (1.31, 5.17)	<.0001
No. unmet needs	0 Needs	1321 (49.41)	643 (48.95)	678 (50.15)	.21
	1 Need	622 (22.16)	292 (21.69)	330 (22.91)	
	2 Needs	343 (11.64)	157 (11.38)	186 (12.06)	
	≥3 Needs	484 (16.79)	240 (17.98)	244 (14.88)	
Gender	Female	140 (5.43)	77 (6.42)	63 (3.84)	.0031
Ethnicity	Hispanic/Latino	117 (4.50)	91 (6.29)	26 (1.63)	<.0001
Race	Black	405 (13.06)	185 (16.96)	220 (6.83)	<.0001
	White	2085 (84.59)	1016 (80.35)	1069 (91.37)	
	Other	276 (2.35)	129 (2.69)	147 (1.80)	
Education	High school or less	1043 (37.14)	441 (32.67)	602 (44.31)	<.0001
Priority group	1	1066 (37.10)	516 (38.72)	550 (34.51)	.14
	2–3	582 (21.19)	286 (20.88)	296 (21.70)	
	4–5	813 (29.92)	373 (28.71)	440 (31.87)	
	6–8	309 (11.78)	157 (11.69)	152 (11.92)	
CCI comorbidity	0	848 (32.23)	406 (31.86)	442 (32.82)	.26
	1	690 (24.53)	321 (23.73)	369 (25.82)	
	≥2	1232 (43.24)	605 (44.42)	627 (41.36)	
Region	Continental	505 (16.98)	245 (15.42)	260 (19.48)	<.001
	Northeast	904 (34.32)	434 (33.72)	470 (35.27)	
	Pacific	451 (14.45)	212 (16.33)	239 (11.44)	
	Southeast	910 (34.25)	441 (34.53)	469 (33.81)	

*SD*, standard deviation; *IQR*, inter quartile range (P25, P75); *CCI*, Charlson Comorbidity Index

The overall burden of social needs was similar between rural and urban Veterans. For example, roughly half (50% and 49% respectively) reported one or more social needs (Table 1). Highly prevalent specific needs were related to finance (22%), food (22%), and legal issues (17%) (Supplemental Table 3). Rural compared to urban Veterans were less likely to report housing needs (6% vs. 10% respectively; *p* < 0.001).

More than half of Veterans (55%) had at least 1 no-show visit and approximately 36% fell below the 80% PDC threshold and were considered medication non-adherent. Urban compared to rural Veterans were more likely to have had a no-show (58% vs. 51%; *p* = 0.002).

### Multivariate Results for No-Show Appointments

The number of visits was related to the risk of having a no-show (OR = 1.11, CI 1.07, 1.15) (Table 2). We found a graded relationship between number of social needs and no-shows with increasing OR values starting at 1.27 (CI 1.01, 1.59); 2.02 (CI 1.49, 2.75); and 3.27 (CI 2.43, 4.39) for one, two, and three or more social needs, respectively. No-show visits were more likely among Black compared to White Veterans (OR = 1.63, CI 1.17, 2.26); they were also more likely among Veterans having multiple comorbidities (OR = 1.71, CI 1.37–2.15), being in priority group 1 (OR = 1.85, CI 1.34, 2.55) or priority groups 4 and 5 (OR = 1.65, CI 1.19, 2.28) compared to priority groups 6 and 7, and residing in the Pacific (OR = 1.43, CI 1.05, 1.94) or Southeast (OR = 1.44, CI 1.15, 1.80) compared to Northeast regions. Results from the sensitivity analysis that adjusted the threshold percentage of missed appointments required to be coded as 1 were consistent with our results using a single binary variable (see Supplemental Table 5).

**Table 2:** Factors Predicting Medication Non-adherence and No-Show Visits

	Medication non-adherence	No-show visits
	OR (95% CI)	OR (95% CI)
*No. Veterans*	*n* = *2,076*	*n* = *2,623*
Age/years	.98 (.97–.99)	.99 (.98–1.00)
No. medications	.93 (.88–.99)	- -
No. visits	- -	1.11 (1.07–1.15)
No. unmet needs (ref = 0)		
1 Needs	.98 (.75–1.28)	1.27 (1.01–1.59)
2 Need	1.09 (.78–1.52)	2.02 (1.49–2.75)
≥3 Needs	1.59 (1.19–2.13)	3.27 (2.43–4.39)
Female	.65 (.39–1.07)	.80 (.50–1.29)
Hispanic/Latino	2.61 (1.56–4.37)	1.52 (.94–2.47)
Race (ref = White)		
Black	1.83 (1.31–2.54)	1.63 (1.17–2.26)
Other	1.73 (1.08–2.75)	1.25 (.81–1.94)
≤High school	1.04 (.83–1.29)	.98 (.81–1.19)
Priority group (ref = groups 6–8)		
Group 1	.88 (.60–1.30)	1.85 (1.34–2.55)
Groups 2–3	1.19 (.79–1.80)	1.38 (.98–1.93)
Groups 4–5	1.19 (.81–1.76)	1.65 (1.19–2.28)
CCI comorbidity (ref = 0)		
1	.94 (.70–1.27)	1.29 (1.00–1.65)
≥2	.99 (.75–1.30)	1.71 (1.37–2.15)
Region (ref = Northeast)		
Continental	.80 (.58–1.09)	.88 (.67–1.15)
Pacific	1.10 (.79–1.53)	1.43 (1.05–1.94)
Southeast	.78 (.60–1.02)	1.44 (1.15–1.80)
Rural	1.05 (.86–1.29)	.89 (.74–1.07)

*OR*, odds ratio; *CI*, confidence interval for OR; *CCI*, Charlson Comorbidity Index

**Figure 1:**
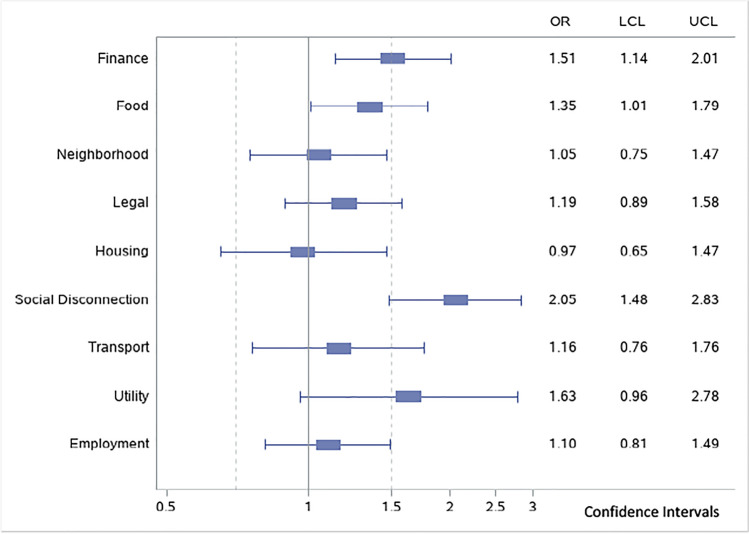
Adjusted odds ratios for individual unmet social needs. Odds ratio represents relationship between no-show visits and specific unmet social need controlling for all other unmet social needs and covariates. OR, odds ratio; LCL, lower confidence limit; UCL, upper confidence limit.

Adjusted models that included each need as a dichotomous variable showed that three of the nine social needs were significantly associated with no-show appointments (Fig. 1). The largest OR was for social disconnection (OR = 2.05, CI 1.48, 2.83) followed by financial needs (OR = 1.51, CI 1.14, 2.01) and food needs (OR = 1.35, CI 1.01, 1.79).

### Multivariate Results for Medication Non-Adherence

**Figure 2:**
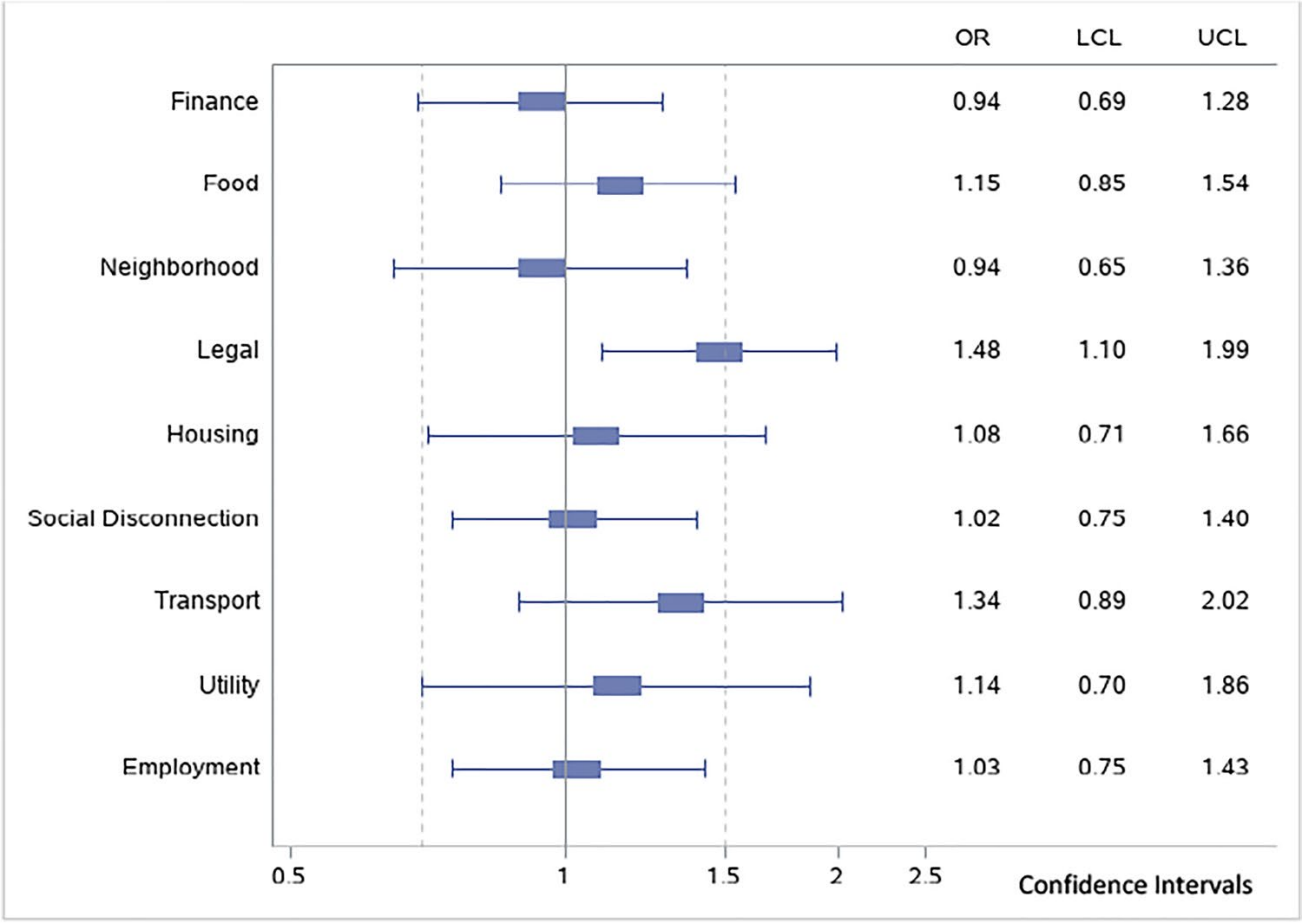
Adjusted odds ratios for individual unmet social needs. Odds ratio represents relationship between medication non-adherence and specific unmet social need controlling for all other unmet social needs and covariates. OR, odds ratio; LCL, lower confidence limit; UCL, upper confidence limit.

Veterans with three or more social needs were more likely to have medication non-adherence (OR = 1.59, CI 1.19, 2.13) (Table 2). The number of medications was inversely related to non-adherence (OR = 0.93, CI 0.88, 0.99) as was age (OR = 0.98, CI 0.97, 0.99). Increased odds of medication non-adherence was associated with Black (OR = 1.83; CI 1.31, 2.54) and other races (OR = 1.73, CI 1.08, 2.75) compared to White Veterans, and Hispanic compared to non-Hispanic Veterans (OR = 2.61, CI 1.56, 4.37). In adjusted models controlling for all unmet needs, only legal needs were significantly associated with a greater likelihood of medication non-adherence (OR = 1.48, CI 1.10, 1.99) (Fig. 2).

### Rurality Effect

Comparing separate rural and urban Veteran multivariate analyses, all CIs for the predictor of interest (unmet need count) were overlapping (Supplemental Table 4). The relationship between  ≥3 unmet social needs and medication non-adherence was significant in the urban model (OR = 1.73, CI 1.16, 2.56) but not in the rural model. A graded relationship between social need burden and no-show visits was observed in both the rural and urban models with a slightly larger OR estimates for urban Veterans with  ≥3 needs (OR = 3.60, CI 2.34, 5.46) compared to their counterpart rural Veterans (OR = 3.03, CI 2.01, 4.57). Conversely, the relationship between having two needs and no-shows was stronger for rural compared to urban Veterans (OR = 2.81, CI 1.87, 4.22 and OR = 1.63, CI 1.05, 2.53, respectively).

## CONCLUSIONS

There are several important findings from this work. First, consistent with our expectation, we found that social need burden was significantly associated with no-shows and medication non-adherence. These findings are consistent with the literature but also expand upon it by providing, we believe for the first time, a study based on a national random sample and with respect to medication adherence, use of an objective measure, and examination of multiple simultaneous social needs.^17,22^

One possible explanation for our findings derives from research demonstrating that increased levels of social needs are associated with stress and adverse attitudes toward preventive and self-care.^44^ Related are studies showing that life chaos (i.e., variability of daily routine, inability to plan and anticipate future activities) affects adherence to recommended care.^45,46^ Thus, the experience of having social needs may present disruptive and competing demands that diminish the ability to access healthcare services needed to achieve the best possible health outcomes. Our findings of the relationship between social needs and care access also provide evidence for potential intermediate outcomes of healthcare-based unmet need interventions (i.e., no-shows and medication adherence). While the long-term goal of such interventions is to improve health outcomes and health equity, assessing intermediate outcomes related to care access could signal in the near-term whether an intervention is moving the needle in the right direction.

Second, we found that the relationship between social needs and care access varied by social need and across study outcome measures. For no-show appointments, the strongest association was for Veterans who endorsed social disconnection. This is somewhat counter to prior studies that report strong associations between no-shows and transportation needs primarily.^13,17–19,47^ However, the relationship between social disconnection and transportation needs is well-established.^48^ Also possible is that the timing of our study—coinciding with the COVID-19 pandemic, a period defined by increased social isolation and barriers to care—helps to explain the strong relationship between social disconnection and no-shows.^49–51^

For medication non-adherence, legal need was the strongest predictor. This is not consistent with prior studies suggesting the influence of housing instability and food needs.^22,52^ At the same time, Veterans’ legal needs are often associated with homelessness and access to benefits and services needed to manage post-traumatic stress disorder or military sexual trauma.^53^ Thus, legal needs may be a signal for an especially vulnerable Veteran population that is by extension at increased risk for medication non-adherence. It is also possible that our findings differ from the literature because a mail survey to capture social needs biased our sample toward Veterans who are stably housed.

Third, somewhat counter to expectations, we found minimal but noteworthy differences between rural and urban Veterans. Across both groups, there was a graded relationship between social needs and no-shows. However, the effect size of having two social needs on no-shows was larger for rural compared to urban Veterans. This may reflect the unique challenges that rural Veterans experience accessing care (longer travel distances, more logistics in planning) such that “lower thresholds” of unmet need burden can more substantially derail efforts to attend medical appointments. We also found that social needs were associated with increased risk for medication non-adherence for urban but not rural Veterans. This merits further inquiry to understand the conditions that moderate the relationship between social needs and medication non-adherence between these groups.

Our study has strengths and limitations. Strengths include the high response rate and use of stratified sampling from broad regions in the USA. Limitations include our focus on Veterans with and at risk for CVD, which may limit generalizability to Veterans without these conditions. However, given the prevalence of these conditions among Veterans, our findings represent a large and important sub-population. Also, for select measures, our analysis assumes that a Veterans endorsement of a social need occurring at the time of the survey is a proxy for the presence of the social need throughout the study period. As well, our sample may be biased toward Veterans with less severe social needs given our use of a mail survey. Finally, the timing of our data collection and analysis (concurrent with the COVID-19 pandemic, a period marked by increased social needs and care access barriers), may have influenced some study results.

In sum, findings suggest that social needs are associated with poor care access. This finding could inform how healthcare-based unmet need interventions are designed and evaluated. Findings also point to specific social needs that may be especially impactful and thus might be prioritized for interventions.

## Supplementary Information

Below is the link to the electronic supplementary material.

Supplementary file1 (DOCX 43 KB)
