# Supplementary material for: Relationship Between Unmet Social Needs and Care Access in a Veteran Cohort
Source: J Gen Intern Med. 2023 Jun 20;38(Suppl 3):841–8. doi: 10.1007/s11606-023-08117-3 (PMC10356706; doi:10.1007/s11606-023-08117-3)
Supplement: Supplementary file 1 — Supplementary file1 (DOCX 43 KB) [file 11606_2023_8117_MOESM1_ESM.docx]

**Supplemental File 1**

**Sample and Sample Weights**

Step 1: We used VA administrative data to select the sampling frame; specific files included outpat.visit, outpat.workload, inpat.inpatient. Eligible patients were those with cardiovascular disease (CVD) or risk of CVD. Patients with CVD were defined by diagnoses of coronary artery disease (I20-I25), cerebrovascular disease (I160-I69), and/or peripheral artery disease (I70-I72). Patients at-risk for CVD were defined by diagnoses of hypertension (I10-I16), diabetes (E08-E13), or hyperlipidemia (E78).

Step 2: We computed a power analysis to estimate the minimum sample size needed for a logistic model. Based on the prior studies, we assumed the baseline rates of medication adherence 30%, and no-shows 5-10% and equal size in rural and urban groups. An adjustment was made since a multiple regression of the rural/urban status on the other variables in the logistic regression obtained an R-Squared of 0.1. With these assumptions and adjustments, we projected a minimum sample size N=1472 in total to have at least 80% power to detect the odds ratio between the two group as small as 1.6 for Medication adherence and no-shows, even when we run the low end of the estimation in baseline rates.” Assuming a 35% response rate, this yielded a survey sample of roughly 4300.

Step 3: We created population sub-groups based on our key strata: race (Black, White, Other), region (Continental, Northeast, Pacific, and Southeast) and cohort (CVD vs. CVD-risk) for a total of 24 strata. We determined the sample from each strata calculated as the proportion of the stratum’s size relative to the population applied to 4300. For example, for strata=1 (race=1, region=1, and cardio=1), the percentage in population is 18.2%, so, the estimated size for this stratum is N=817. Strata sample size ranged from N=5 to N=817.

Step 4: We oversampled small strata to improve the precision of our estimates. Assuming a 35% response rate, we increased small sample strata to n=86, the minimum size needed to yield a final sample of n= 30 (the minimum sample size needed to ensure a sufficient sample size to conduct a statistical inference based on a normal distribution). This yielded a final sample of 5,204 Veterans to survey.

Step 4: Sample weights were derived as 1/selection probability. The selection probability was the sample size for that stratum divided by the population of that stratum.

**Supplemental Table 1.**

**Drug Classifications (and example medications) used to Examine Medication Non-adherence**

Ace inhibitors (ramiripil, lisinopril, benazepril)

Angiotensin II inhibitors (irbesartan, candesartan, telmisartan)

Antianginals (Isosorbude dinitrate, nitroglycerin, diltiazem)

Antilipemic agents (pravastatin, fenobiric acid, rosuvastatin)

Beta blockers and related drugs (propranolol, carvedilol, labetalol)

Calcium channel blockers (amlodipine, nifedipine, verapamil)

Carbonic anhydrase inhibitor diuretics (acetazolamide, methazolamide)

Digitalis glycosides (digoxin)

Direct renin inhibitors (losartan, enalapril)

Hypoglycemic agents (liraglutide, glimepiride)

Loop diuretics (bumetanide, furosemide, torsemide)

Nicotinic acid (niacin, niacinamide)

Oral hypoglycemic agents (metformin, glyburide, glipizide)

Platelet aggregation inhibitors (clopidogrel, ticagrelor, dipyridamole)

Potassium sparing and combination diuretics (spironolactone, triamterene)

Thiazides and related diuretics (hydralazine, chlorthalidone, indapamide)

**Supplemental Table 2. Survey questionnaire**

| **Item** | **Question** | **Coding for analysis** | **Source** | **Domain** |
| --- | --- | --- | --- | --- |
| 1 | In which U.S. Military branch(es) did you serve? (Check all that apply.)   - Army - Air Force - Coast Guard - Navy - Marine Corps - Other:__________________ | N/A; not used in analysis | Adapted from [Veterans and Active Duty Military Survey](https://www.battlefields.org/veterans-and-active-duty-military-survey); changed from “Which Military branch did you serve with” | Military branch |
| 2 | What is your living situation today? (Check only one.)   - I have a steady place to live - I have a place to live today, but I am worried about losing it in the future - I do not have a steady place to live *(ex. I am temporarily staying with others, in a hotel, in a shelter, living outside on the street, on a beach, in a car, abandoned building, bus or train station, or in a park)* | 1= I have a place to live today, but I am worried about losing it in the future OR I do not have a steady place to live  0 = I have a steady place to live | AHC HRSN^1^ | Housing instability |
| 3 | Which of the following types of people currently live with you in your household? (Check all that apply.)   - My spouse or partner - My child(ren) under age 18 - My adult child(ren) age 18 or older - My parent(s) - Extended Family - Roommate(s) not related to me - Other individuals | N/A; not used in analysis | Adapted from [Report on the Economic Well-Being of U.S. Households (SHED)](https://www.federalreserve.gov/publications/2019-appendix-a-survey-questionnaire.htm); replaced “do each” with “which of the” | Household |
| 4 | In the past 12 months has the electric, gas, oil or water company threatened to shut off service in your home?   - Yes - No - Already shut off - Not applicable | 1 = Yes OR Already shut off  0 = No OR Not applicable | Adapted from AHC HRSN^1^; added “Not applicable” as a response option | Utilities |
| 5 | Indicate how well you agree with the following statement: *“My neighborhood is a safe place to live.”* (Check only one.)   - Strongly Disagree - Disagree - Mostly Disagree - Neither Agree nor Disagree - Mostly Agree - Agree - Strongly Agree | 1 = Strongly Disagree, Disagree, OR Mostly Disagree  0 = Neither Agree nor Disagree, Mostly Agree, Agree, OR Strongly Agree | [Roots to Health Survey](http://www.thecivicengine.org/opensource.html) | Neighborhood safety |
| 6 | What is your current work situation? (Check only one.)   - Full time work - Part-time or temporary work - Unemployed - Otherwise unemployed but not seeking work *(ex. Student, retired, disabled, unpaid primary care giver)* | 1 = Unemployed  0 = Full time, Part-time, OR Otherwise unemployed | Adapted from PRAPARE^2^; removed “I choose not to answer this question” response option | Employment |
| 7 | In the past 12 months, has lack of reliable transportation kept you from medical appointments, meetings, work or from getting things you need for daily living?   - Yes - No | 1 = Yes  0 = No | AHC HRSN^1^ | Transportation |
| 8 | In the past 12 months, have you felt you needed or wanted help for legal problems?   - Yes - No | 1 = Yes  0 = No | Adapted from [Black Rural and Urban Caregivers Mental Health/Functioning, Missouri, 1999-2002](https://www.icpsr.umich.edu/icpsrweb/ICPSR/studies/36349/datasets/0001/variables/E167?archive=icpsr); changed from “Have you felt you needed or wanted help in the past six months for Legal problems?” | Legal needs |
| 9 | Overall, which one of the following best describes how well you are managing financially these days?   - Living comfortably - Doing ok - Just getting by - Finding it difficult to get by - Prefer not to answer | 1 = Just getting by OR Finding it difficult to get by  0 = Living comfortably, Doing ok, OR Prefer not to answer | [Report on the Economic Well-Being of U.S. Households (SHED)](https://www.federalreserve.gov/publications/2019-appendix-a-survey-questionnaire.htm) | Financial needs |
| 10a & 10b | Within the past 12 months, you worried that your food would run out before you got money to buy more.   - Never True - Sometimes True - Often True   Within the past 12 months, the food you bought just didn't last and you didn't have  money to get more.   - Never True - Sometimes True - Often True | 1 = Sometimes OR Often to either item  0 = Never to both items | AHC HRSN^1^ | Food insecurity |
| 11 | In a typical week, how many times do you talk on the telephone, or by video (ex. Skype, Facetime, etc.), with family, friends or neighbors? [Open response field] | N/A; not used in analysis | Adapted from Berkman-Syme Social Network Index | Social isolation |
| 12 | In a typical week, how often do you get together with friends or relatives? [Open response field] |  |  |  |
| 13 | In the past year, how often did you attend church or religious services? [Open response field] |  |  |  |
| 14 | Do you belong to any clubs or organization such as a church group, union, fraternal or athletic group, or school group?   - Yes - No |  |  |  |
| 15 | How often do you feel lonely or isolated from those around you?   - Never - Rarely - Sometimes - Often - Always | 1 = Often OR Always  0 = Never, Rarely, OR Sometimes | Adapted from [English Longitudinal Study of Ageing](https://www.ageuk.org.uk/globalassets/age-uk/documents/reports-and-publications/reports-and-briefings/loneliness/180917_loneliness-report-technical-report_final.pdf); changed from “How often do you feel lonely?” Changed response options from 3 to 5 point scale. | Social Disconnection |
| 16 | If for any reason you need help with day-to-day activities such as bathing, preparing meals, shopping, managing finances, etc., do you get the help you need? (Check only one.)   - I don’t need any help - I get all the help I need - I could use a little more help - I need a lot more help | 1 = I could use a little more OR I need a lot more  0 = I didn’t need any OR I get all the help I need | AHC HRSN^1^ | Help with daily activities |
| 17 | What is the highest level of school that you have finished?   - Less than high school degree - High school diploma or GED - More than high school | 1 = More than high school  0 = Less than high school OR High school diploma or GED | PRAPARE^2^ | Education |
| 18 | Are you of Hispanic or Latino origin or descent?   - Yes, Hispanic or Latino - No, Not Hispanic or Latino | 1 = Yes  0 = No | VA SHEP^3^ | Ethnicity |
| 19 | What is your race? (Mark one or more.)   - White - Black or African American - Asian - Native Hawaiian or other Pacific Islander - American Indian or Alaska Native | 1 = White  2 = Black  0 = Asian, Native Hawaiian or other Pacific Islander, OR American Indian or Alaska Native | VA SHEP^3^ | Race |
| 20 | In general, how would you rate your overall health?   - Poor - Fair - Good - Very good - Excellent | N/A; not used in analysis | Adapted from [RAND 36-Item Short Form Survey Instrument (SF-36)](https://www.rand.org/health-care/surveys_tools/mos/36-item-short-form.html); changed from In general, would you say your health is: | Self-Rated Health |
| 21 | Were you infected by COVID-19?   - Yes, confirmed by a health professional - I experienced symptoms, but a diagnosis was not confirmed - No - Do not know | N/A; not used in analysis | [Health Care Worker COVID-19 Survey](https://publichealth.gwu.edu/covidsurvey) | COVID-19 |
| 22 | We may follow-up with some survey participants to see if they would like to participate in another study. The potential study would gather more in-depth information about Veterans’ health and social service needs. Could we contact you again to ask if you are interested in participating?   - Yes - No | N/A; not used in analysis | Created by study team | Permission to be recontacted |

^1^Accountable Health Communities Heath-Related Social Needs (AHC HRSN) <https://innovation.cms.gov/Files/worksheets/ahcm-screeningtool.pdf>

^2^Protocol for Responding to and Assessing Patients’ Assets, Risks and Experiences (PRAPARE) <http://www.nachc.org/wp-content/uploads/2018/05/PRAPARE_One_Pager_Sept_2016.pdf>

^3^Survey of Healthcare Experiences of Patients (SHEP) <https://vaww.qps.med.va.gov/divisions/api/pm/shep/shepDefault.aspx>

**Supplemental Table 3.**

**Unmet Social Needs Among Rural and Urban Veterans**

|  | **Overall**  **n (%)** |  | **Urban**  **n (%)** |  | **Rural**  **n (%)** |  | ***P*-value** |
| --- | --- | --- | --- | --- | --- | --- | --- |
|  | **(n=2,770)** |  | **(n=1,332)** |  | **(n=1,438)** |  |  |
| Finance | 639 (22.60) |  | 299 (22.37) |  | 340 (22.97) |  | .72 |
| Food | 642 (21.73) |  | 299 (22.03) |  | 343 (21.25) |  | .63 |
| Neighborhood | 242 (8.55) |  | 123 (9.26) |  | 119 (7.40) |  | .09 |
| Legal | 502 (17.02) |  | 240 (17.59) |  | 262 (16.10) |  | .32 |
| Living | 231 (8.45) |  | 126 (9.96) |  | 105 (6.02) |  | <.001 |
| Social Disconnection | 398 (14.07) |  | 207 (15.14) |  | 191 (12.35) |  | .04 |
| Transport | 195 (7.00) |  | 103 (7.79) |  | 92 (5.75) |  | .04 |
| Utility | 149 (5.60) |  | 77 (5.78) |  | 72 (4.44) |  | .13 |
| Work | 311 (11.04) |  | 148 (11.38) |  | 163 (10.49) |  | .47 |
|  | | | | | | | |

**Supplemental Table 4.**

**Factors Predicting Medication Non-Adherence and No-Show Visits for Rural and Urban Veterans**

|  | **Medication non-adherence** | | | **No-show visits** | |
| --- | --- | --- | --- | --- | --- |
|  | **Urban** | | **Rural** | **Urban** | **Rural** |
|  | **OR (95% CI)*** | | **OR (95% CI)** | **OR (95% CI)** | **OR (95% CI)** |
| ***No. Veterans*** | | ***n=2,076*** | | ***n=2,623*** | |
| Age - years | | .98 (.96 - .99) | .99 (.97 - 1.01) | .99 (.97 - 1.00) | 1.00 (.98 - 1.01) |
| No. Medications | | .91 (.84 - .99) | .96 (.89 - 1.04) | - - | - - |
| No. Visits | | - - | - - | 1.15 (1.10 - 1.21) | 1.07 (1.03 - 1.11) |
| No. Unmet Needs (Ref = 0) | |  |  |  |  |
| 1 Need | | .93 (.64 - 1.35) | 1.05 (.72 - 1.51) | 1.35 (.98 - 1.87) | 1.13 (.83 - 1.55) |
| 2 Needs | | 1.18 (.73 - 1.91) | .97 (.62 - 1.50) | 1.63 (1.05 - 2.53) | 2.81 (1.87 - 4.22) |
| ≥3 Needs | | 1.73 (1.16 - 2.56) | 1.43 (.95 - 2.17) | 3.60 (2.34 - 5.46) | 3.03 (2.01 - 4.57) |
| Female | | .70 (.38 - 1.32) | .47 (.22 - 1.02) | .76 (.41 - 1.41) | .82 (.42 - 1.60) |
| Hispanic/Latino | | 2.64 (1.4- 4.75) | 2.88 (.96 - 8.66) | 1.50 (.86 - 2.61) | 1.62 (.57 - 4.65) |
| Race (Ref = White) | |  |  |  |  |
| Other | | 2.05 (1.09 - 3.86) | 1.18 (.71 - 1.96) | 1.19 (.66 - 2.15) | 1.50 (.92 - 2.45) |
| Black | | 1.83 (1.21 - 2.78) | 1.77 (1.15 - 2.72) | 1.75 (1.14 - 2.67) | 1.21 (.79 - 1.82) |
| ≤ High School | | 1.10 (.81 - 1.50) | .92 (.68 - 1.23) | 1.02 (.77 - 1.36) | .91 (.70 - 1.18) |
| Priority Group (Ref = Grp 6-8) | |  |  |  |  |
| Group 1 | | .80 (.47 - 1.37) | 1.03 (.59 - 1.79) | 1.63 (1.05 - 2.53) | 2.04 (1.28 - 3.26) |
| Groups 2 - 3 | | 1.15 (.65 - 2.05) | 1.31 (.73 - 2.35) | 1.38 (.86 - 2.19) | 1.30 (.80 - 2.13) |
| Groups 4 - 5 | | 1.10 (.64 - 1.90) | 1.41 (.81 - 2.47) | 1.51 (.96 - 2.36) | 1.80 (1.12 - 2.89) |
| CCI Comorbidity^†^ (Ref = 0) | |  |  |  |  |
| 1 | | .82 (.54 - 1.25) | 1.13 (.76 - 1.67) | 1.45 (1.01 - 2.08) | 1.07 (.77 - 1.49) |
| ≥2 | | 1.07 (.73 - 1.55) | .89 (.61 - 1.30) | 1.97 (1.44 - 2.70) | 1.38 (1.00 - 1.89) |
| Region (Ref = Northeast) | |  |  |  |  |
| Continental | | .79 (.51 - 1.23) | .76 (.49 - 1.18) | .95 (.65 - 1.41) | .79 (.54 - 1.15) |
| Pacific | | 1.02 (.65 - 1.59) | 1.24 (.80 - 1.93) | 1.31 (.87 - 1.98) | 1.70 (1.12 - 2.58) |
| Southeast | | .73 (.51 - 1.05) | .86 (.60 - 1.23) | 1.40 (1.01 - 1.92) | 1.57 (1.15 - 2.14) |
| *OR = odds ratio - CI = confidence interval for OR - ^†^Charlson Comorbidity Index (CCI) | | | | | |

| **Supplemental Table 5. Factors Predicting No-Shows Visits: Sensitivity Analysis Based on No-Show Rate Cut Points** | | |  |  |
| --- | --- | --- | --- | --- |
|  | **0 vs. >.00 to 10%** | **0 vs. >10% to 33%** | **0 vs. >33% to 55%** | **0 vs. >55%** |
|  | **OR (95% CI)*** | **OR (95% CI)** | **OR (95% CI)** | **OR (95% CI)** |
| ***No. Veterans*** | ***n=108*** | ***n=572*** | ***n=529*** | ***n=275*** |
| Age/years | 1.01 (.98 - 1.04) | 1.00 (.99 - 1.01) | .99 (.98 - 1.00) | .98 (.96 - .99) |
| No. Visits | 1.34 (1.27 - 1.43) | 1.25 (1.19 - 1.32) | 1.01 (.96 - 1.06) | .75 (.67 - .85) |
|  |  |  |  |  |
| No. Unmet Needs (Ref = 0) |  |  |  |  |
| 1 Needs | 1.23 (.62 - 2.44) | .84 (.60 - 1.16) | 1.55 (1.14 - 2.11) | 1.67 (1.11 - 2.52) |
| 2 Need | 1.91 (.85 - 4.33) | 1.66 (1.12 - 2.46) | 2.17 (1.46 - 3.23) | 2.82 (1.70 - 4.68) |
| ≥3 Needs | 2.68 (1.29 - 5.57) | 2.12 (1.41 - 3.18) | 3.85 (2.69 - 5.51) | 5.07 (3.25 - 7.91) |
|  |  |  |  |  |
| Female | .27 (.05 - 1.40) | .59 (.28 - 1.24) | 1.15 (.66 - 2.01) | .79 (.34 - 1.82) |
|  |  |  |  |  |
| Hispanic/Latino | 1.20 (.45 - 3.19) | .81 (.40 - 1.66) | 1.15 (.89 - 3.09) | 2.87 (1.45 - 5.56) |
|  |  |  |  |  |
| Race (Ref = White) |  |  |  |  |
| Black | .94 (.38 - 2.33) | 1.21 (.77 - 1.91) | 1.84 (1.24 - 2.73) | 2.44 (1.49 - 4.00) |
| Other | 1.32 (.42 - 4.21) | 1.23 (.70 - 2.18) | 1.44 (.86 - 2.41) | .81 (.44 - 1.52) |
| ≤ High School |  |  |  |  |
|  | .86 (.51 - 1.46) | 1.01 (.77 - 1.32) | .92 (.71 - 1.20) | 1.04 (.74 - 1.46) |
|  |  |  |  |  |
| Priority Group (Ref = Grp 6-8) |  |  |  |  |
| Group 1 | 1.19 (.51 - 2.77) | 1.45 (.94 - 2.22) | 1.91 (1.17 - 3.10) | 4.08 (1.95 - 8.56) |
| Groups 2 - 3 | .64 (.24 - 1.71) | .90 (.57 - 1.43) | 1.93 (1.17 - 3.17) | 2.23 (1.03 - 4.82) |
| Groups 4 - 5 | .75 (.29 - 1.92) | 1.22 (.79 - 1.88) | 2.03 (1.25 - 3.30) | 3.00 (1.44 - 6.26) |
|  |  |  |  |  |
| Comorbidity^†^ (Ref = 0) |  |  |  |  |
| 1 | 1.25 (.61 - 2.55) | 1.48 (1.05 - 2.09) | 1.33 (.96 - 1.85) | 1.06 (.67 - 1.69) |
| ≥2 | 1.17 (.63 - 2.18) | 1.72 (1.26 - 2.34) | 1.60 (1.19 - 2.16) | 2.56 (1.71 - 3.84) |
|  |  |  |  |  |
| Region (Ref = Northeast) |  |  |  |  |
| Continental | 1.19 (.54 - 2.63) | 1.01 (.70 - 1.46) | .78 (.54 - 1.15) | .85 (.53 - 1.39) |
| Pacific | 1.88 (.84 - 4.24) | 1.42 (.96 - 2.12) | 1.42 (.95 - 2.13) | 1.31 (.77 - 2.23) |
| Southeast | 2.05 (1.09 - 3.88) | 1.42 (1.04 - 1.94) | 1.39 (1.03 - 1.86) | 1.36 (.91 - 2.04) |
|  |  |  |  |  |
| Rural | .58 (.34 - .98) | .85 (.66 - 1.09) | .91 (.71 - 1.15) | .94 (.69 - 1.29) |
| *OR = odds ratio, CI = confidence interval for OR, ^†^Charlson Comorbidity Index (CCI) | | |  |  |
